# Microclimatic responses to shelterbelt structural configuration in an arid cotton agroforestry system

**DOI:** 10.3389/fpls.2026.1859047

**Published:** 2026-07-16

**Authors:** Xiaoqian Li, Ping Lv, Zhuo Zhang, Lanjie Li, Xue Yang, Zhengli Zhou, Yawen Wang, Kaiwen Tan, Cheng Tang, Qiang Jin, Ruiqi Zhang

**Affiliations:** 1 College of Urban and Environmental Sciences, Shihezi University, Shihezi, China; 2 College of Agriculture, Shihezi University, Shihezi, China; 3 Xinjiang Production and Construction Corps Forestry and Grassland Work Station, Urumqi, China; 4 College of Horticulture and Forestry, Tarim University, Alar, China

**Keywords:** agroforestry system, cotton growth stages, farmland shelterbelt, microclimate regulation, shelterbelt structure configuration

## Abstract

**Introduction:**

Farmland shelterbelts serve as important ecological barriers that protect agricultural systems. Investigating the effects of shelterbelts with different structural configurations on farmland microclimate is of great significance for optimizing shelterbelt structure and improving farmland ecological stability.

**Methods:**

In this study, structural characteristics including the number of rows, length, spacing, and porosity were comprehensively considered. Three types of shelterbelt configurations (Grade I, II, and III) were selected using a typical sampling method in the 12th Regiment of Alar City, Xinjiang, with open farmland serving as the control (CK). Microclimate factors, including wind speed (WS), air temperature (TA), air humidity (RH), light intensity (LI), and soil temperature (ST), were measured at different horizontal distances from the shelterbelt during four cotton growth stages (Seedling Stage, Budding Stage, Flowering Stage, Boll Opening Stage).

**Results:**

The results showed that the microclimate-regulating effect of shelterbelts increased with the improvement of shelterbelt grade, generally following the order Grade I > Grade II > Grade III. Among them, Grade I shelterbelts showed the most pronounced wind-speed reduction and hydrothermal regulation effects throughout the whole cotton growth period. The relative wind-speed reduction rate ranged from 72.50% to 97.78%, while the relative interaction index (RII) ranged from −0.164 to 0.010 for LI, from −0.063 to 0.166 for RH, from −0.055 to 0.005 for ST, and from −0.066 to 0.012 for TA. Spatially, wind speed under all three shelterbelt grades reached its minimum at 0.5H and then gradually increased with distance from the shelterbelt. TA was relatively low at 0.5H, and the cooling effect was more pronounced at noon. RH was higher at 1H and gradually approached the control level with increasing distance. LI increased with height in the vertical direction, while its horizontal distribution was relatively stable, with only a slight decrease near the shelterbelt. ST at the 10–15 cm soil layer was lower than that at the 0–10 cm layer, and a low-temperature center occurred horizontally at 2H–3H, with ST generally decreasing as the cotton growth period progressed.

**Discussion:**

This study indicates that shelterbelt structures with more rows, better belt continuity, richer tree species composition, and moderate porosity are more conducive to improving the farmland microclimate. These findings provide a theoretical basis for optimizing farmland shelterbelt structure and regulating microclimate in agroforestry systems in arid regions.

## 1 Introduction

Shelterbelts, as an important ecological barrier in China’s Three-North Shelterbelt Project (Jiao-jun and Xiao, 2019), are closely related to wind and sand control, soil and water conservation, and microclimate regulation (Cao et al., 2020; Qi et al., 2023; Zhai et al., 2023). By blocking wind and sand erosion, they form wind-speed reduction zones within the protected areas of shelterbelts, thereby producing a series of chain effects, including reducing turbulence and thus stabilizing wind speed; altering the radiation balance, lowering soil temperature and improving humidity; enhancing evapotranspiration, increasing local humidity, and improving soil water retention capacity (Guo et al., 2020, 2014; Van Thuyet et al., 2014).

In farmland ecosystems, microclimate factors—including air temperature, relative humidity, solar radiation intensity, wind speed, and soil temperature—play a crucial role (Yang et al., 2021). Their combined effects not only determine crop growth and development but also reflect the distribution of farmland resources, thus exerting significant influences on crop growth (Kowalchuk and deJong, 1995; Mattera et al., 2013; Kong et al., 2023). Studies have shown that farmland shelterbelts improve farmland microclimate conditions, including water, light, heat, and soil resources, thereby further affecting crop yield (den Herder et al., 2017; Laroche et al., 2019). However, the overall protective effectiveness of shelterbelts is determined not only by stand-scale structure but also by shelterbelt structural configuration (Jiaojun et al., 2003). Previous studies (Zhaomin et al., 1981) have found that excessively small shelterbelt networks increase the area affected by wind erosion, resulting in poor airflow and imbalanced temperature and humidity, which are unfavorable for crop growth; conversely, excessively large networks weaken ecological protection effects. The number of rows in shelterbelts is directly related to their microclimate regulation capacity (Cui et al., 2012). In the Ulan Buh Desert region, a four-row shelterbelt configuration exhibits significant effects in wind reduction, increasing air humidity, and stabilizing surface temperature (Hongli et al., 2009). In coastal shelterbelts, larger canopy gaps have a more significant impact on microclimate than smaller ones (Xie et al., 2024). Studies on shelterbelts in paddy fields in western Jilin Province indicate that shelterbelt permeability has an important influence on temperature, humidity, and rice yield within the sheltered area (Shan-shan et al., 2015).

In addition, the effects of shelterbelts on farmland microclimate exhibit significant spatiotemporal variability. Potashkina and Koshelev (2022) found that shelterbelts exert different regulatory effects on microclimate during different seasons and crop growth stages. Studies have shown that, with the progression of the cotton growth period, air temperature and light intensity first increase and then decrease (Wang et al., 2022). In the Hetao Irrigation District, soil moisture within farmland shelterbelt networks is most significantly affected within a horizontal distance of one tree height (1H) from both sides of the shelterbelt (Liyuan et al., 2025). Therefore, analyzing the effects of shelterbelts with different structural configurations on the spatiotemporal variation of microclimate is of great significance for improving the sustainability of agroforestry ecosystems and maintaining ecological balance. Although previous studies have demonstrated that different shelterbelt configurations significantly affect the spatiotemporal variation of farmland microclimate, there is still a lack of research on the classification of shelterbelt structural configurations and on how different levels of shelterbelts influence farmland microclimate.

Typical sampling is a non-probability sampling technique (Etikan and Bala, 2017). By selecting representative samples, it ensures that the selected samples can reflect the characteristics of the whole research object. The purpose of this method is to ensure that the sampled shelterbelts can fully represent the diversity within the study area, thereby obtaining results with strong representativeness. From an ecological perspective (Stoutjesdijk and Barkman, 2015) emphasized that microclimate is the “minor weather” in the surrounding environment of plants and animals. Its formation is closely related to the interactions among topography, soil, vegetation, and organisms, and different habitats and species may have their own specific microclimatic conditions (Bramer et al., 2018). considered microclimate to generally refer to the local climate within tens of meters around plants or stands, with a spatial scale of <100 m. Based on the above views, this study mainly focuses on the regulation of the near-surface farmland environment by shelterbelts. Wind speed, light intensity, air temperature, air humidity, and soil temperature were selected as the core indicators of microclimate variation. Meanwhile, the observation range in this study was set at 0.5–5H in the horizontal direction (H = 15 m) and 30–90 cm in the vertical direction.

Therefore, this study comprehensively considered shelterbelt structural characteristics, including the number of rows, length, plant spacing, and porosity. Using a typical sampling method, farmland shelterbelts located in the 12th Regiment of Alar were selected, and their structural configurations were classified into three grades, namely Grade I, Grade II, and Grade III shelterbelts. Farmland microclimate during different cotton growth seasons, including air temperature, air humidity, soil temperature, wind speed, and light intensity, was monitored and studied to analyze the variation patterns of the effects of different grades of farmland shelterbelts on farmland microclimate. The results provide a theoretical basis and scientific foundation for the scientific configuration of shelterbelt structure and regional ecological protection. To better understand the regulatory mechanism of shelterbelts on farmland microclimate, we hypothesized that: (1) shelterbelt structural configuration has a significant effect on microclimate regulation, and the farmland microclimate regulation functions, including wind reduction, shading, cooling, and humidification, will be significantly enhanced with the improvement of shelterbelt structural configuration quality; (2) the microclimatic effects of shelterbelts show an obvious spatial attenuation pattern; and (3) the response of microclimate to shelterbelt structural configuration differs among different cotton growth stages.

## 2 Materials and methods

### 2.1 Study area overview

**Figure 1:**
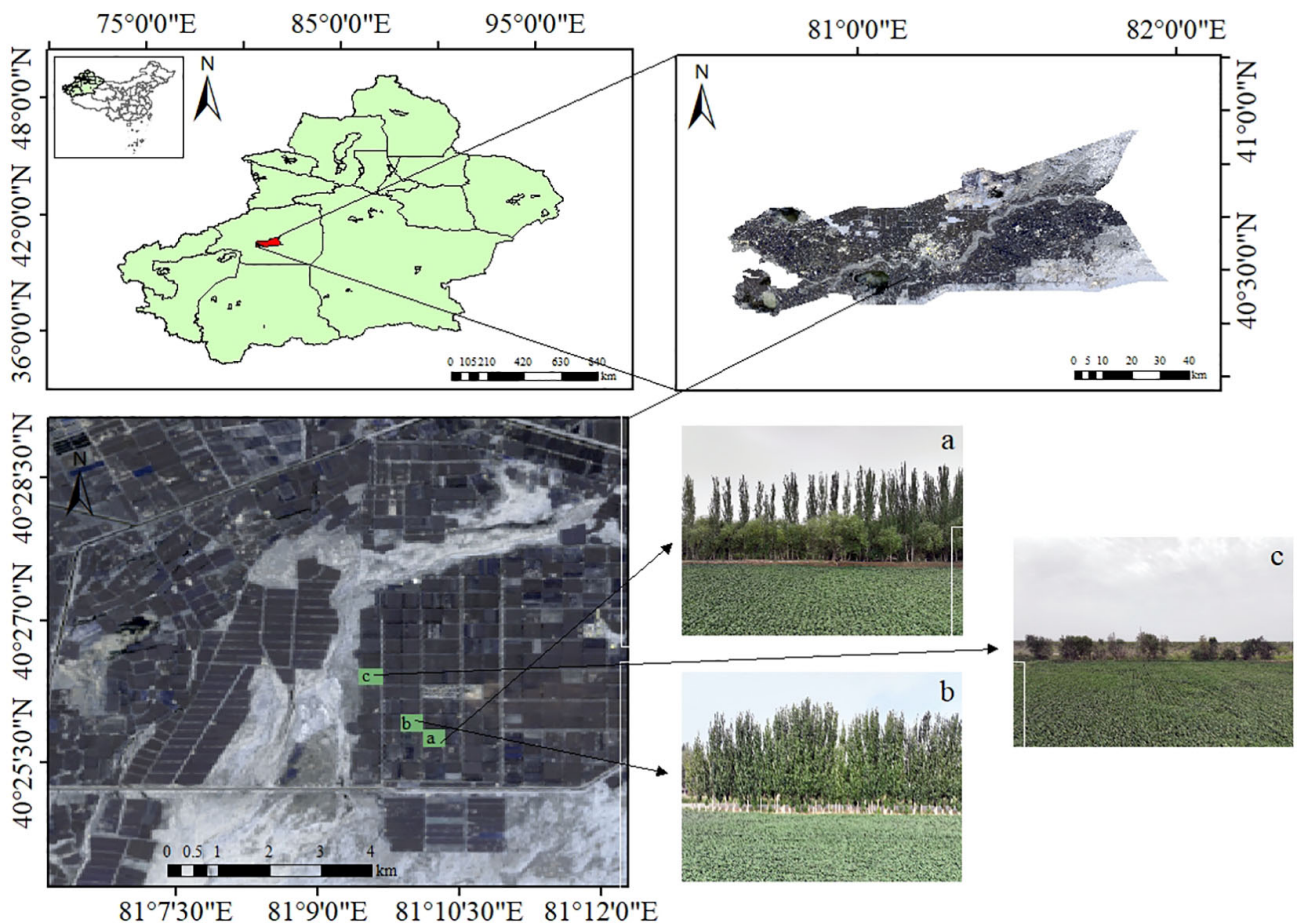
Location of the study area and structural configuration of farmland shelterbelts with different grades. **(a)** Grade I shelterbelt, **(b)** Grade II shelterbelt, **(c)** Grade III shelterbelt.

The study area is located in the Alar Reclamation Area, situated on the northwestern edge of the Taklamakan Desert (40°43’-40°59’N, 81°16’-81°57’E) (**Figure 1**). It has a typical temperate continental climate with scarce precipitation, ranging from 40 to 82 mm annually, while the average annual evaporation is 1800–2500 mm. Strong winds and sandstorms occur frequently. The region is rich in light and heat resources but experiences large temperature variations, with an extreme maximum temperature of 45 °C and an extreme minimum temperature of -28 °C. The average annual solar radiation in the reclamation area is 133.7–146.3 kcal/cm², the annual sunshine duration ranges from 2556.3 to 2991.8 hours, and the sunshine percentage is 58.69% (Ling et al., 2025; Shengru et al., 2025). Based on several structural indicators, including shelterbelt row number, shelterbelt length, tree height, diameter at breast height (DBH), spacing between plants and rows, porosity, and shelterbelt continuity, the farmland shelterbelts in the study area were classified into three grades: Grade I, Grade II, and Grade III. The basic information is shown in **Table 1**.

**Table 1:** Basic structural characteristics of farmland shelterbelts with different grades.

Farmland shelterbelt grade	Direction	Length(m)	Number of rows	Tree species	Average tree height(m)	DBH(m)	Average tree spacing(m)	Average row spacing(m)	Average branch height(m)	Porosity(%)
I	East forest belt	270	3	*Populus alba* var. *pyramidalis* Bge*., Populus euphratica* Oliv.	19.5	0.3	1.8	2	2	35
	South forest belt	300	2	*Populus alba* var. *pyramidalis* Bge*., Populus euphratica* Oliv.	18.5	0.3	1.8	2	2.1	40
	West forest belt	270	4	*Populus alba* var. *pyramidalis* Bge*., Populus euphratica* Oliv*., Tamarix chinensis* Lour.	17.5	0.25	1.8	2	1.8	30
	North forest belt	300	3	*Populus alba* var. *pyramidalis* Bge*., Populus euphratica* Oliv.	18.0	0.3	1.8	2	2	35
II	East forest belt	280	3	*Populus alba* var. *pyramidalis* Bge.	17	0.25	1.5	1.5	1.8	45
	South forest belt	250	2	*Populus alba* var. *pyramidalis* Bge*., Populus euphratica* Oliv.	15	0.3	1.5	1.5	1.5	40
	West forest belt	280	1	*Populus euphratica* Oliv.	13.5	0.25	1.5	Broken belt	1.5	55
	North forest belt	250	2	*Populus alba* var. *pyramidalis* Bge*., Populus euphratica* Oliv.	15	0.25	1.5	1.5	1.8	40
III	East forest belt	290	1	*Populus alba* var. *pyramidalis* Bge.	11.5	0.15	1.5	Broken belt	1.5	70
	South forest belt	260	1	*Populus euphratica* Oliv.	10	0.2	1.5	Broken belt	1.2	70
	West forest belt	290	1	*Populus euphratica* Oliv.	10.5	0.25	1.5	Broken belt	1.2	60
	North forest belt	260	1	*Populus alba* var. *pyramidalis* Bge.	11.5	0.2	1.5	Broken belt	1.5	65

Grade I, Grade II, and Grade III represent farmland shelterbelts with different structural configurations, classified according to row number, belt length, tree height, DBH, plant and row spacing, porosity, and belt continuity. Direction indicates the position of each shelterbelt around the farmland. Tree species indicates the main species composition of each shelterbelt. Length, average tree height, average tree spacing, average row spacing, and average branch height are expressed in m. DBH refers to diameter at breast height and is expressed in m. Porosity (%) represents the degree of shelterbelt openness. “Broken belt” indicates that the shelterbelt was discontinuous or incomplete in that direction.

### 2.2 Plot setup and survey plot design and field investigation

In this study, the structural characteristics of shelterbelts, including the number of rows, length, plant spacing, and porosity, were comprehensively considered. Using a typical sampling method, three farmland shelterbelts with different structural configurations, namely Grade I, Grade II, and Grade III shelterbelts, were selected in the 12th Regiment of Alar City, Xinjiang (**Table 1**), with unsheltered farmland used as the control. The shelterbelts were all in the mature stage, and the main crop planted was cotton.

**Figure 2:**
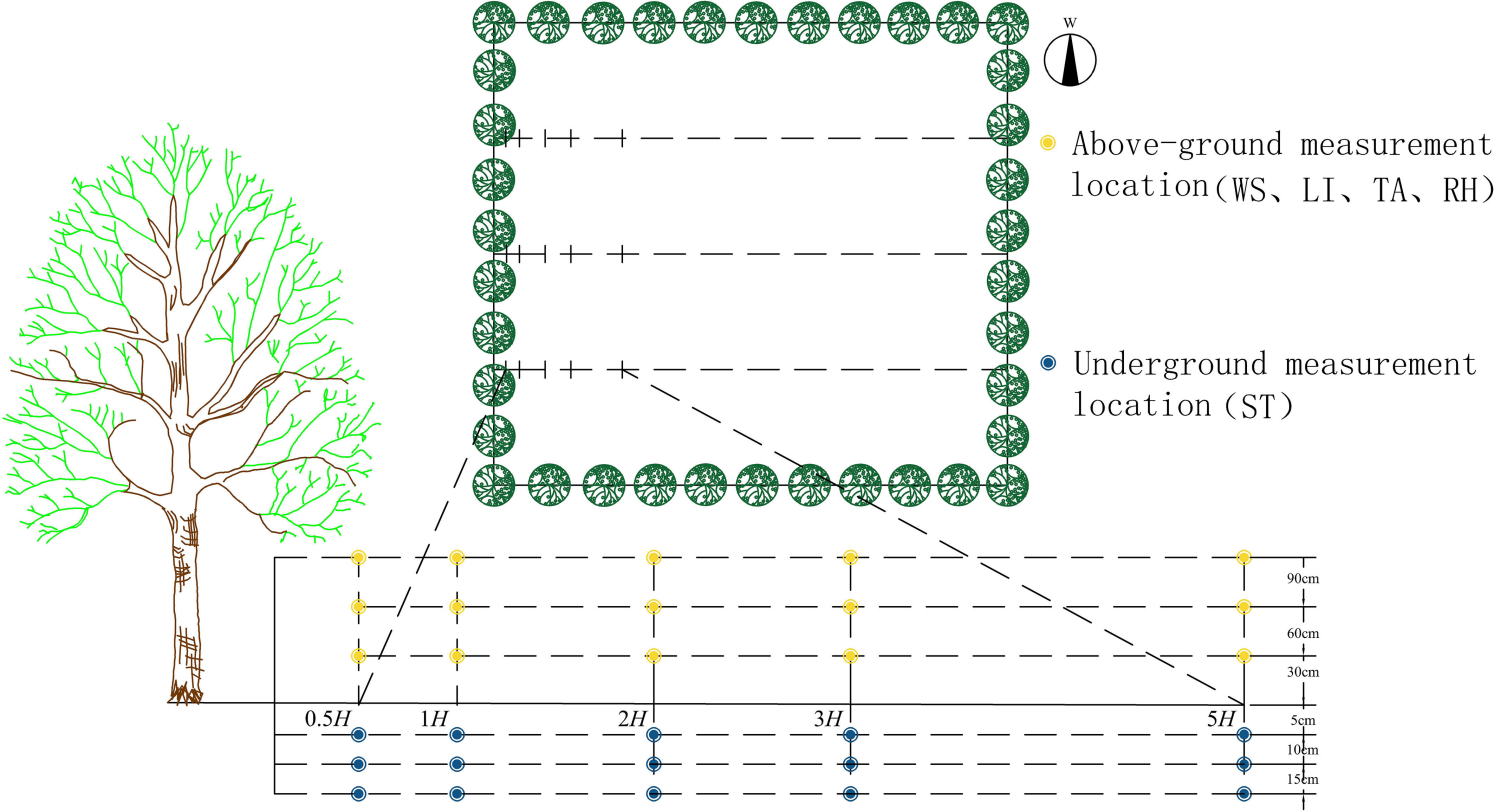
Schematic diagram of microclimate measurement points in farmland shelterbelts. WS denotes wind speed, LI denotes light intensity, TA denotes air temperature, RH denotes air humidity, and ST denotes soil temperature.

To study field microclimate changes during the crop growing season, the farmland was divided into plots to ensure that each experimental plot under different shelterbelt configurations had uniform treatments, such as fertilization, irrigation, and other field management measures. Wind speed (WS) was measured using a portable anemometer and wind direction meter (PLC-16025, Beijing, China; wind speed accuracy ±3%, wind direction accuracy ±3°). Light intensity (LI) was measured using a handheld spectral illuminance meter (HP320, Hangzhou, China; measurement accuracy ±5%). Air temperature (TA), air humidity (RH), and soil temperature (ST) were measured using a temperature and humidity data logger (S21AN, Xuzhou, China; temperature accuracy ±0.3°C, humidity accuracy ±3%, soil temperature accuracy ±0.5 °C). All sensors were calibrated before installation, and the measured data were obtained from instantaneous measurements repeated three times and then averaged. Specifically, data were collected continuously for 3 days during the cotton seedling stage (June 8), budding stage (July 14), flowering stage (August 27), and boll-opening stage (October 5). Measurements were conducted at the same time in the morning, noon, and evening under representative weather conditions, and farmland without shelterbelts under the same conditions was selected as the natural control group for microclimate monitoring within the shelterbelt network. The main shelterbelt was the southern shelterbelt. Along the main shelterbelt and perpendicular to the shelterbelt direction, three equally spaced transects were arranged according to the farmland area. Monitoring points were set at 0.5H, 1H, 2H, 3H, and 5H along each transect (**Figure 2**), where H represents the mean shelterbelt height. Together with the control farmland, a total of 18 monitoring points were established. Wind speed, air temperature and humidity, and light intensity were recorded at heights of 30, 60, and 90 cm, and soil temperature was measured at depths of 5, 10, and 15 cm from the ground surface. To ensure temporal consistency of the data from each plot, measuring instruments were placed at each monitoring point in the three grades of farmland shelterbelts and in the unsheltered control area (CK), and microclimate data were monitored simultaneously at the same time to reduce data errors. No rainfall or irrigation occurred within 10 days before sampling to exclude the effects of irrigation and rainfall.

### 2.3 Calculation methods for microclimate factors

#### 2.3.1 Relative interaction index

The relative interaction index (RII) was used to characterize the regulatory effect of shelterbelts on farmland microclimate factors. This index has good symmetry and standardization characteristics (Armas et al., 2004).


$$R_{II}=\frac{U-Uck}{U+Uck}$$

In the formula, U represents the measured data at each measurement point behind the shelterbelt, and Uck represents the measured data under control conditions.

In this study, both the measured values of microclimate factors and the relative interaction index were reported because they reflect different information. The measured values were used to describe the actual magnitude of microclimate factors at different distances and within different grades of shelterbelts, for example, air temperature expressed in °C, air humidity expressed in %, light intensity expressed in Lux, and soil temperature expressed in °C. The relative interaction index (RII) was used to eliminate the influence of differences in background microclimate conditions among different observation periods and to characterize the relative regulatory intensity of shelterbelts compared with the unsheltered control area (CK). RII is a dimensionless index. RII > 0 indicates that the factor within the shelterbelt was higher than CK, RII < 0 indicates that it was lower than CK, and RII = 0 indicates that it was basically consistent with CK. Therefore, the measured values and RII were used to describe the absolute levels and relative regulatory effects of microclimate factors, respectively.

#### 2.3.2 Relative wind speed reduction rate *R*


$$R=(1-\frac{U}{\text{U}})\times 100\%$$

In the formula, *R* represents the relative wind speed reduction rate at each measuring point; *U′* represents the wind speed at each measuring point (m/s); and *U* represents the open field control wind speed (m/s) (Cornelis and Gabriëls, 2005).

Due to differences in wind speed among measurement periods under field observation conditions, the absolute wind speed values among different growth stages were not directly comparable. Therefore, in this study, the R value was defined as the “relative wind-speed reduction rate” and was used to describe the effect efficiency of shelterbelts relative to the reference background microclimate, rather than the absolute effect efficiency.

#### 2.3.3 Statistical analysis

Basic data processing and statistical analyses were completed using Excel 2020 and SPSS 27.0 (IBM SPSS, Armonk, NY, USA). Normality and homogeneity of variance tests were conducted for air temperature (TA) and air humidity (RH) data using SPSS 27.0 to meet the basic assumptions of one-way analysis of variance. On this basis, one-way ANOVA was used to test the significance of differences in air temperature and air humidity among different horizontal distances. When the ANOVA results reached a significant level, Duncan’s multiple range test was used for *post hoc* multiple comparisons, with the significance level set at P < 0.05. Wind speed (WS), light intensity (LI), and soil temperature (ST) were mainly used to describe the spatial distribution characteristics and variation patterns under different shelterbelt structural configurations.

To comprehensively evaluate the effects of shelterbelts with different structural configurations on farmland microclimate, principal component analysis (PCA) was used to simultaneously integrate multiple variables, including WS, TA, RH, LI, and soil temperature at different soil depths, namely ST5 (5 cm), ST10 (10 cm), and ST15 (15 cm). Because the variables had different units, all variables were standardized before PCA. Data graphs were drawn using Origin 2024 and Python software, and wind-speed flow field maps were completed using Surfer 28. Kriging interpolation was used for spatial interpolation analysis to characterize the spatial distribution characteristics of wind speed.

## 3 Results

### 3.1 Wind speed

#### 3.1.1 Statistical characteristics of wind speed variation within the shelterbelt network

In terms of shelterbelt grade (**Table 2**), Grade I shelterbelts showed the strongest wind-speed reduction capacity, with a relative wind-speed reduction rate of 72.5% to 97.78%. Grade II shelterbelts ranged from 62.25% to 78.89%, while Grade III shelterbelts showed the lowest reduction capacity, reaching 76.78% only at the seedling stage and decreasing to 20.75% and 30.22% at the flowering stage and boll-opening stage, respectively. In terms of spatial distribution, shelterbelts of all grades showed a consistent pattern across different growth stages. Wind speed was lowest at 0.5H and 1H, and gradually increased with increasing horizontal distance. In terms of cotton growth stages, the overall wind speed during the budding stage was relatively low, and wind speed at 0.5H and 1H under Grade I shelterbelts was 0 during this stage. Wind speed was relatively high at the seedling stage, while wind speed and relative wind-speed reduction rate fluctuated to some extent at the flowering and boll-opening stages. However, the overall performance of Grade I shelterbelts was better than that of Grade II and Grade III shelterbelts. In addition, the average wind speed within all shelterbelt grades was lower than that of CK, and the relative wind-speed reduction rates were all positive, indicating that farmland shelterbelts had an obvious wind-speed reduction effect.

**Table 2:** Average wind speed at different horizontal distances and relative wind-speed reduction within farmland shelterbelts of different grades.

Farmland shelterbelt grade	Measurement period	Wind speed (m·s^-^¹) at different horizontal distances (H)						CK	R/%
		0.5H	1H	2H	3H	5H	Average		
I	Seedling Stage	0.02	0.07	0.35	0.53	0.72	0.34	1.67	79.81
	Budding Stage	0.00	0.00	0.03	0.06	0.09	0.04	1.60	97.78
	Flowering Stage	0.03	0.04	0.08	0.09	0.12	0.07	0.27	72.50
	Boll Opening Stage	0.00	0.01	0.09	0.12	0.21	0.09	0.50	82.67
II	Seedling Stage	0.12	0.26	0.33	0.49	0.61	0.36	1.67	78.38
	Budding Stage	0.00	0.19	0.42	0.50	0.58	0.34	1.60	78.89
	Flowering Stage	0.02	0.02	0.04	0.18	0.24	0.10	0.27	62.25
	Boll Opening Stage	0.04	0.07	0.12	0.21	0.48	0.18	0.50	63.11
III	Seedling Stage	0.04	0.11	0.33	0.63	0.82	0.39	1.67	76.78
	Budding Stage	0.33	0.50	0.67	0.60	1.13	0.65	1.60	59.58
	Flowering Stage	0.11	0.13	0.18	0.24	0.39	0.21	0.27	20.75
	Boll Opening Stage	0.10	0.16	0.20	0.59	0.70	0.35	0.50	30.22

#### 3.1.2 Spatial variation of wind speed during different growth stages

**Figure 3:**
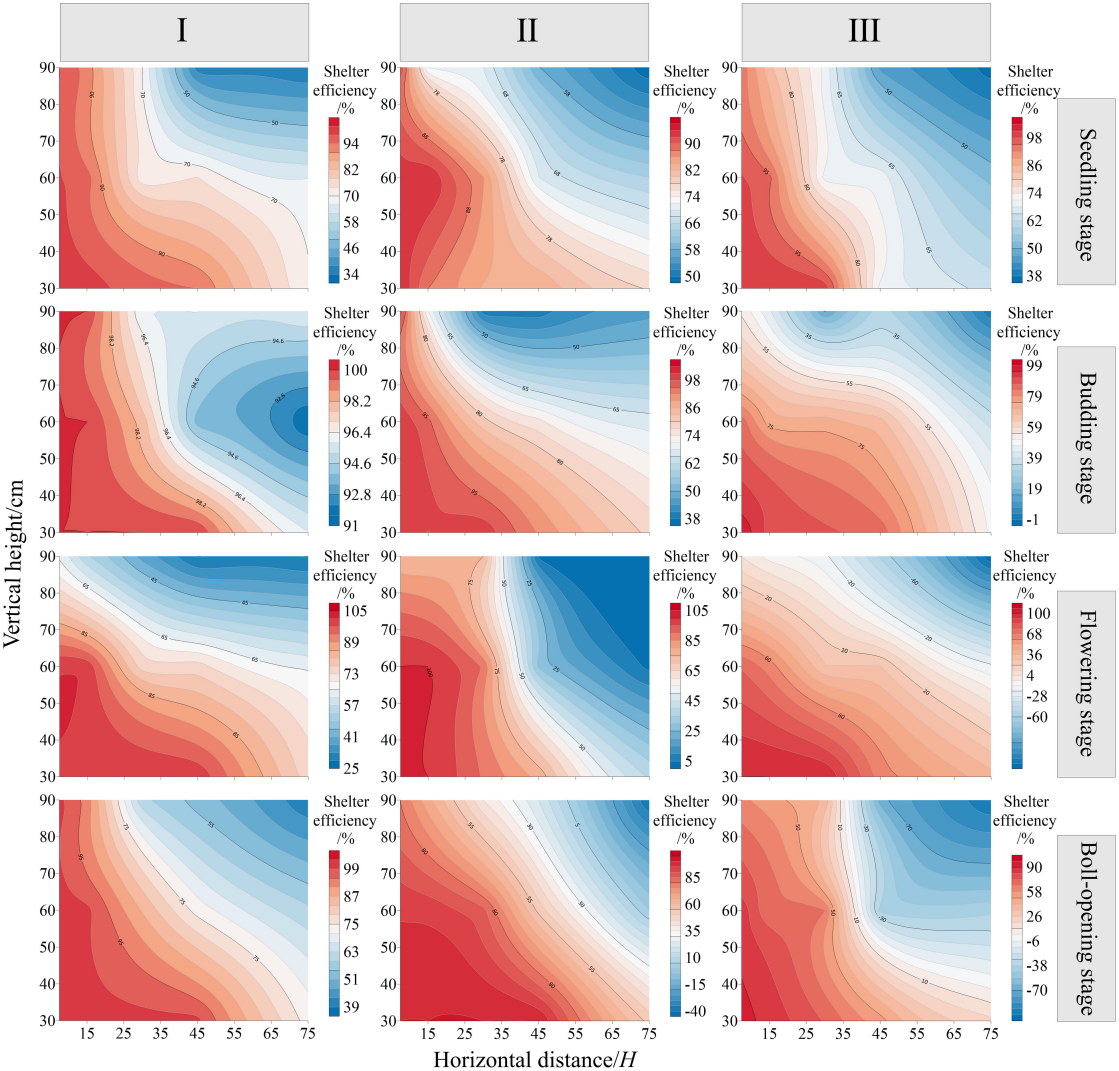
Spatial variation of wind speed with horizontal distance and measurement height within farmland shelterbelts of different grades.

As shown in **Figure 3**, the relative wind speed reduction rates differed obviously among shelterbelts of different grades. Overall, Grade I shelterbelts exhibit the strongest wind reduction capacity, followed by Grade II, while Grade III shows the weakest effect, indicating that a better shelterbelt structural configuration corresponds to a stronger wind reduction effect. In terms of spatial distribution, the area near the shelterbelt at 0.5H–1H serves as the core wind reduction zone. As the horizontal distance increases to 2H, the reduction effect gradually weakens, and the contour lines shift from dense to sparse, indicating a gradual recovery of wind speed. Vertically, the wind reduction effect is strongest at heights of 30 cm and 60 cm, and gradually weakens with increasing height, suggesting that airflow at upper layers is less affected by the shelterbelts. In addition, the effective influence range of Grade I shelterbelts is distinctly larger than that of Grade II and Grade III, with its reduction effect extending to a greater distance, whereas the influence of Grade III shelterbelts is mainly limited to the area near the shelterbelt. Regarding cotton growth stages, the overall wind reduction effect is strongest during the Budding Stage, with the largest extent of high-value zones, suggesting that the combined effect of shelterbelts and crop canopy structure produces the most pronounced airflow retardation during this period. The reduction effect is relatively weak at the Seedling Stage, with a gentle spatial gradient. Fluctuations are observed during the Flowering Stage and Boll Opening Stage, with weakened or even negative reduction effects in some areas, indicating that the regulatory capacity of shelterbelts declines as the cotton growing season progresses.

### 3.2 Light intensity under farmland shelterbelts

#### 3.2.1 Statistical characteristics of LI variation within the shelterbelt network

In terms of shelterbelt grade (**Table 3**), Grade I shelterbelts showed the strongest reduction effect on light intensity, with the RII of LI ranging from −0.164 to −0.046 throughout the whole growth period. For example, the average light intensity at the flowering stage was 20268 Lux, which was lower than that of CK. Grade II shelterbelts ranked second, with RII values ranging from −0.109 to −0.012, while Grade III shelterbelts showed the weakest effect, with RII values ranging from −0.005 to 0.010, and were even slightly higher than CK at the boll-opening stage. In terms of spatial distribution, all shelterbelt grades showed the lowest light intensity at 0.5H, which gradually increased with distance to 1H–2H. Under Grade I shelterbelts at the seedling stage, light intensity increased from 40048 Lux at 0.5H to 45057 Lux at 3H, indicating that the shading effect of the tree canopy was mainly concentrated within the range of 0.5H–2H. However, light intensity did not fully recover within the range of 3H–5H, and even showed lower or higher values in some growth stages. In terms of growth stages, LI was lowest at the flowering stage and increased again at the boll-opening stage. The three shelterbelt grades showed similar trends, but Grade I shelterbelts had the greatest variation amplitude.

**Table 3:** Average light intensity at different horizontal distances and relative interaction index within farmland shelterbelts of different grades.

Farmland shelterbelt grade	Measurement period	Light intensity (Lux) at different horizontal distances (H)						CK	RII
		0.5H	1H	2H	3H	5H	Average		
I	Seedling Stage	40048	42254	43768	45057	43941	43014	54955	-0.122
	Budding Stage	10037	15265	15658	13909	12738	13522	17302	-0.123
	Flowering Stage	18605	19739	20080	19578	23338	20268	28231	-0.164
	Boll Opening Stage	19867	20863	21271	21610	22176	21157	23193	-0.046
II	Seedling Stage	50601	52459	52470	55130	57649	53662	54955	-0.012
	Budding Stage	14917	15634	15687	15405	15822	15493	17302	-0.055
	Flowering Stage	19664	21439	24360	23675	24242	22676	28231	-0.109
	Boll Opening Stage	21189	22237	22205	22443	21483	21912	23193	-0.028
III	Seedling Stage	53152	55702	53800	54934	54849	54487	54955	-0.004
	Budding Stage	16124	16965	17537	17760	17309	17139	17302	-0.005
	Flowering Stage	26269	27575	29101	29395	28651	28198	28231	-0.001
	Boll Opening Stage	22667	23328	23790	24691	23929	23681	23193	0.010

#### 3.2.2 Spatial variation of LI within the shelterbelt network

**Figure 4:**
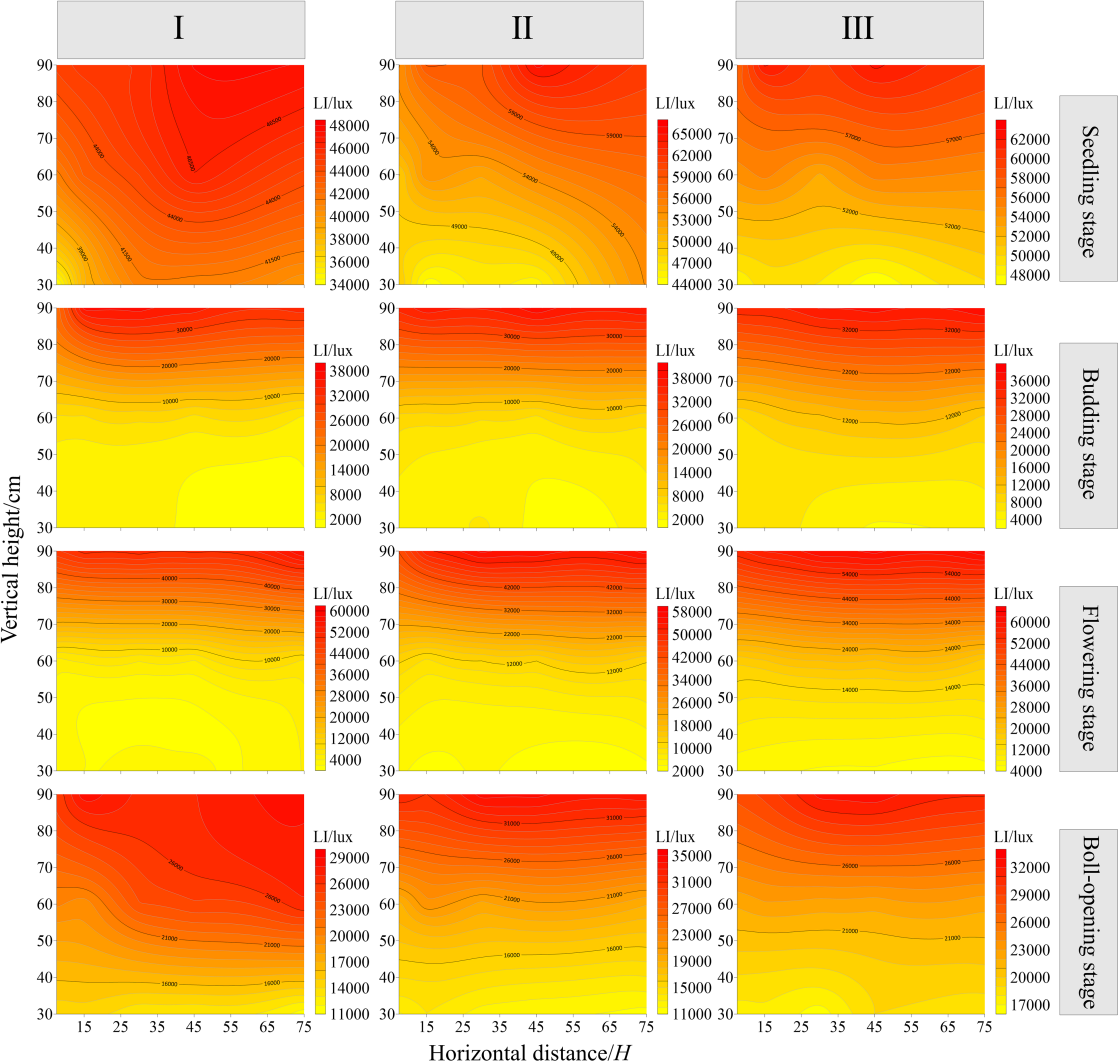
Spatial variation of light intensity with horizontal distance and measurement height within farmland shelterbelts of different grades.

In terms of shelterbelt grade (**Figure 4**), shelterbelts of different grades differed in their regulation of LI. LI under Grade I shelterbelts was lower than that under Grade II and Grade III shelterbelts in all periods, while Grade III shelterbelts showed relatively higher overall LI. In terms of spatial distribution, LI showed a consistent pattern among all shelterbelt grades. Light intensity was relatively low in the near-shelterbelt area of 0.5H–1H, gradually increased with horizontal distance to 2H, and then tended to become stable, indicating that the direct shading effect of the tree canopy was mainly concentrated within 0.5H–2H. Meanwhile, in the vertical direction, LI increased with height. The shading effect at 90 cm was relatively weak, whereas light intensity in the middle and lower layers at 30–60 cm decreased to some extent, especially during the budding and flowering stages. At the seedling stage, due to the lower plant height, the shading effect of the crop canopy at 60–90 cm was slightly weaker than that in other stages. At the boll-opening stage, the crop was basically mature and senescent, and light transmittance increased, reflecting the combined effects of the crop canopy and shelterbelt.

### 3.3 Soil temperature under farmland shelterbelts

#### 3.3.1 Statistical characteristics of soil temperature variation within the shelterbelt network

Shelterbelts of different grades had an obvious regulatory effect on soil temperature (**Table 4**), generally showing a cooling effect. Grade I shelterbelts showed the greatest reduction in soil temperature, with RII values ranging from −0.055 to −0.029. For example, the average soil temperature at the seedling stage was 28.50 °C, which was lower than that of CK. Grade II shelterbelts ranked second, with RII values ranging from −0.038 to −0.025. Grade III shelterbelts showed a relatively weaker regulatory effect, with RII values ranging from −0.051 to 0.005, and were even slightly higher than CK at the boll-opening stage. In terms of spatial distribution, all shelterbelts showed relatively low temperature at 0.5H near the shelterbelt, which gradually increased with distance to 1H–2H and reached relatively high values within the range of 3H–5H. For example, under Grade II shelterbelts at the seedling stage, soil temperature increased from 26.63 °C at 0.5H to 33.62 °C at 5H. In terms of growth stages, the cooling effect was stronger at the seedling and boll-opening stages, while it was relatively weaker at the budding and flowering stages. Soil temperature under Grade III shelterbelts was close to or even exceeded CK at the flowering and boll-opening stages.

**Table 4:** Average soil temperature at different horizontal distances and relative interaction index within farmland shelterbelts of different grades.

Farmland shelterbelt grade	Measurement period	Soil temperature (°C) at different horizontal distances (H)						CK	RII
		0.5H	1H	2H	3H	5H	Average		
I	Seedling Stage	26.38	28.62	28.81	29.24	29.46	28.50	31.79	-0.055
	Budding Stage	21.60	22.01	22.47	22.21	23.30	22.32	23.63	-0.029
	Flowering Stage	20.46	20.79	20.71	22.61	23.81	21.67	23.32	-0.037
	Boll Opening Stage	15.96	16.60	16.35	16.85	16.85	16.52	18.19	-0.048
II	Seedling Stage	26.63	28.43	30.55	31.45	33.62	30.14	31.79	-0.027
	Budding Stage	21.81	22.33	22.59	22.68	22.98	22.48	23.63	-0.025
	Flowering Stage	21.03	21.12	21.15	21.19	23.62	21.62	23.32	-0.038
	Boll Opening Stage	16.29	16.57	17.16	17.50	17.01	16.91	18.19	-0.037
III	Seedling Stage	27.32	28.20	28.79	29.43	29.77	28.70	31.79	-0.051
	Budding Stage	22.03	24.49	23.44	23.99	22.64	23.32	23.63	-0.007
	Flowering Stage	24.03	25.40	22.79	21.66	22.31	23.23	23.32	-0.002
	Boll Opening Stage	18.44	20.29	17.57	18.20	17.42	18.39	18.19	0.005

#### 3.3.2 Spatial variation of soil temperature within the shelterbelt network

**Figure 5:**
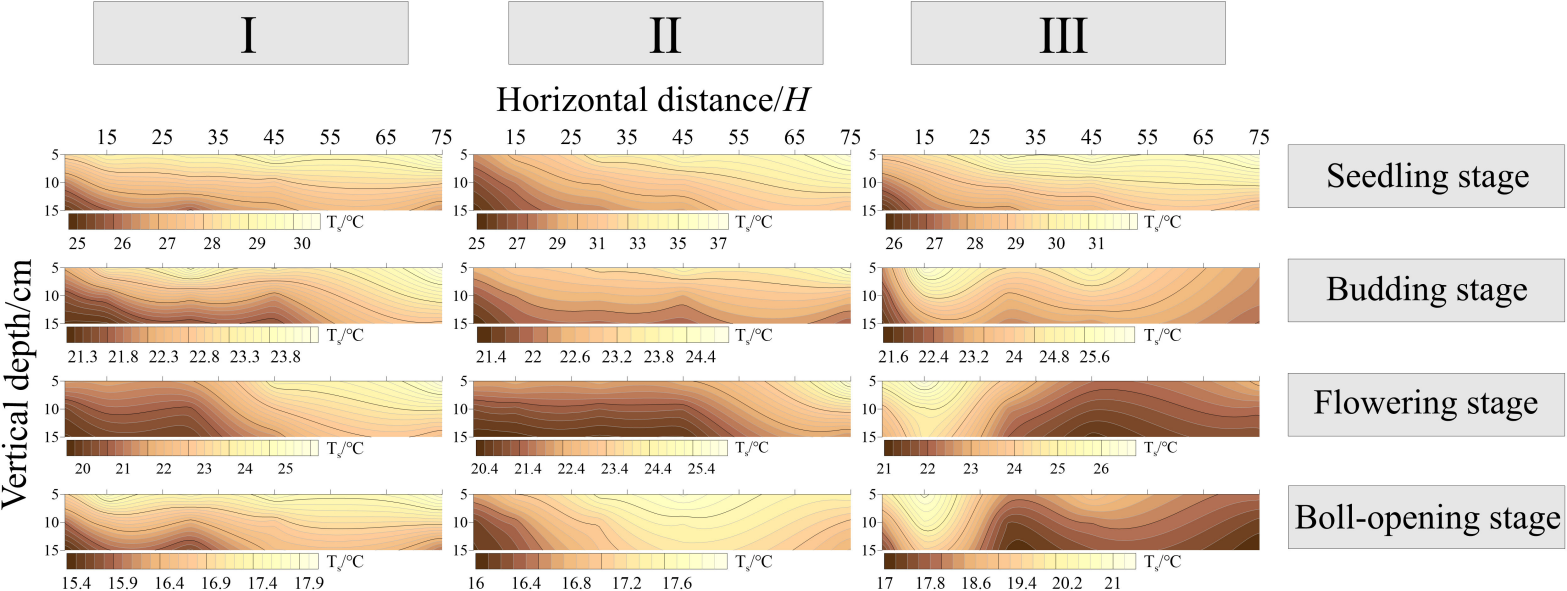
Spatial variation of soil temperature with horizontal distance and soil depth within farmland shelterbelts of different grades.

The regulation of soil temperature by shelterbelts of different grades showed obvious differences in both space and time (**Figure 5**). Grade I shelterbelts showed the most obvious cooling effect, followed by Grade II shelterbelts, while Grade III shelterbelts showed a relatively weaker regulatory effect and even exhibited a warming phenomenon in some periods. In terms of spatial distribution, all shelterbelt grades showed similar patterns. Soil temperature was relatively low in the near-shelterbelt area of 0.5H–1H, gradually increased with horizontal distance to 2H, and then tended to become stable or showed local fluctuations. In terms of vertical distribution, soil temperature was higher in the shallow soil layer at 5 cm, while it decreased and became more stable as soil depth increased to 15 cm. In terms of growth stages, soil temperature was generally higher at the seedling stage, gradually decreased during the budding and flowering stages, and reached the lowest level at the boll-opening stage. Grade I shelterbelts showed more obvious temperature gradients in all periods. In addition, soil temperature in some areas far from the shelterbelt (>2H) under Grade III shelterbelts was higher than that in the near-shelterbelt area, indicating its weaker regulatory capacity.

### 3.4 Air humidity under farmland shelterbelts

#### 3.4.1 Statistical characteristics of air humidity variation within the shelterbelt network

As shown in **Table 5**, the average air humidity under Grade I shelterbelts ranged from 39.47% to 69.69% across different growth stages, which was higher than that under Grade II and Grade III shelterbelts, with RII values ranging from 0.094 to 0.166. The average air humidity under Grade II shelterbelts ranged from 31.98% to 64.57%, with RII values ranging from 0.017 to 0.104. The average air humidity under Grade III shelterbelts was only 27.22%–56.80%, and the RII values were negative at the seedling and boll-opening stages. In terms of spatial distance, air humidity was generally highest at 1H and decreased toward both sides. In terms of growth stages, the humidification effect was most obvious at the budding stage, with the average air humidity under Grade I, Grade II, and Grade III shelterbelts reaching 69.69%, 64.57%, and 56.80%, respectively. The flowering stage ranked second, with the average air humidity of the three grades ranging from 52.76% to 63.18%. Air humidity was relatively low at the seedling and boll-opening stages. At the seedling stage, the average air humidity under Grade I, Grade II, and Grade III shelterbelts was 45.54%, 40.08%, and 32.34%, respectively; at the boll-opening stage, it was 39.47%, 31.98%, and 27.22%, respectively. 

**Table 5:** Average air humidity at different horizontal distances and relative interaction index within farmland shelterbelts of different grades.

Farmland shelterbelt grade	Measurement period	Air humidity (%) at different horizontal distances (H)						CK	RII
		0.5H	1H	2H	3H	5H	Average		
I	Seedling Stage	42.08	51.58	47.69	42.66	43.67	45.54	32.55	0.166
	Budding Stage	67.06	75.62	70.97	67.50	67.32	69.69	54.15	0.126
	Flowering Stage	59.41	67.87	63.85	62.46	62.29	63.18	52.28	0.094
	Boll Opening Stage	38.65	40.72	39.98	38.82	39.21	39.47	30.89	0.122
II	Seedling Stage	37.89	43.62	42.17	39.86	36.84	40.08	32.55	0.104
	Budding Stage	60.27	72.77	65.31	63.73	60.78	64.57	54.15	0.088
	Flowering Stage	52.12	67.36	59.27	55.45	54.81	57.80	52.28	0.050
	Boll Opening Stage	30.18	34.03	32.79	31.78	31.11	31.98	30.89	0.017
III	Seedling Stage	30.54	36.89	33.96	31.24	29.07	32.34	32.55	-0.003
	Budding Stage	52.47	67.23	60.89	53.90	49.49	56.80	54.15	0.024
	Flowering Stage	48.56	60.64	54.56	53.57	46.49	52.76	52.28	0.005
	Boll Opening Stage	25.69	28.28	27.07	27.67	27.41	27.22	30.89	-0.063

#### 3.4.2 Spatiotemporal variation of air humidity within the shelterbelt network

**Figure 6:**
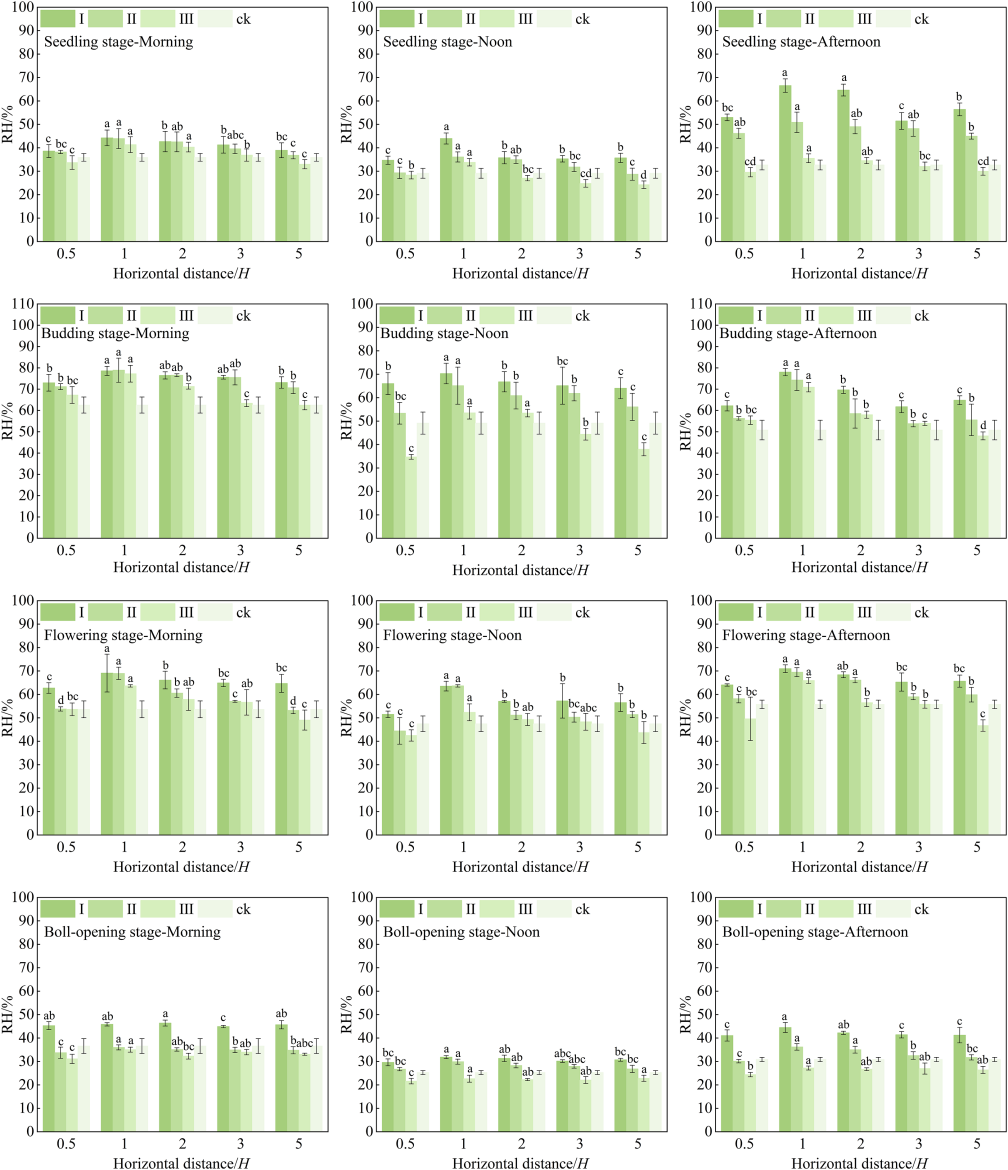
Variation in air humidity with horizontal distance and observation period within farmland shelterbelts of different grades. Different lowercase letters indicate significant differences in air humidity among different horizontal distances within the same shelterbelt grade, growth stage, and observation period (p < 0.05).

In terms of shelterbelt grade (**Figure 6**), Grade I shelterbelts consistently showed the strongest humidification effect. Air humidity under Grade I shelterbelts was generally higher than that under Grade II, Grade III shelterbelts, and CK across different growth stages, observation periods, and spatial distances. Grade II shelterbelts ranked second, while Grade III shelterbelts showed the weakest humidification effect, with little difference from CK at noon during the boll-opening stage. In terms of spatial distance, air humidity was highest at 1H and decreased toward both sides along the horizontal distance. Humidity at 0.5H and 2H was slightly lower than that at 1H, while humidity at 3H and 5H gradually approached the CK level, and the difference between 1H and 5H was relatively obvious (**Table 6**). In terms of growth stages, overall humidity was highest at the budding and flowering stages, followed by the seedling stage, and lowest at the boll-opening stage, with relatively consistent diurnal variation patterns. Among the morning, noon, and evening observation periods, humidity was generally lowest at noon and increased again in the morning and evening. Humidity during all observation periods was relatively low at the boll-opening stage, indicating that the humidification effect of shelterbelts was stronger at the budding and flowering stages but weakened at the boll-opening stage.

**Table 6:** One-way ANOVA results for air humidity among horizontal distances within farmland shelterbelts of different grades.

Measurement period	Statistics	Morning			Noon			Afternoon		
		I	II	III	I	II	III	I	II	III
Seedling Stage	F_(df1, df2)_	F_(4,40)_=4.69	F_(4,40)_=7.62	F_(4,40)_=14.55	F_(4,40)_=23.29	F_(4,40)_=10.42	F_(4,40)_=37.49	F_(4,40)=_37.68	F_(4,40)_=3.11	F_(4,40)_=10.82
	p	<0.003	<0.001	<0.001	<0.001	<0.001	<0.001	<0.001	<0.026	<0.001
Budding Stage	F_(df1, df2)_	F_(4,40)_=2.89	F_(4,40)_=7.97	F_(4,40)_=22.66	F_(4,40)_=3.47	F_(4,40)_=5.105	F_(4,40)_=33.30	F_(4,40)_=9.72	F_(4,40)_=9.02	F_(4,40)_=41.92
	p	<0.034	<0.001	<0.001	<0.016	<0.002	<0.001	<0.001	<0.001	<0.001
Flowering Stage	F_(df1, df2)_	F(_4,40)_=2.77	F_(4,40)_=31.93	F_(4,40)_=8.16	F_(4,40)_=5.55	F_(4,40)_=12.80	F_(4,40)_=2.62	F_(4,40)_=3.57	F_(4,40)_=30.23	F_(4,40)_=6.93
	p	<0.04	<0.001	<0.001	<0.001	<0.001	<0.049	<0.014	<0.001	<0.001
Boll Opening Stage	F_(df1, df2)_	F_(4,40)_=5.72	F(_4,40)_=4.26	F_(4,40)_=4.35	F_(4,40)_=4.89	F_(4,40)_=4.67	F_(4,40)_=3.17	F(_4,40)_=3.10	F_(4,40)_=19.86	F_(4,40)_=2.74
	p	<0.001	<0.006	<0.005	<0.003	<0.003	<0.024	<0.026	<0.001	<0.042

The results indicate the significance of differences in air temperature among horizontal distances (0.5H, 1H, 2H, 3H, and 5H) within the same shelterbelt grade, growth stage, and observation period. F represents the one-way ANOVA statistic, df1 and df2 represent the between-group and within-group degrees of freedom, respectively, and p represents the significance level.

### 3.5 Air temperature under farmland shelterbelts

#### 3.5.1 Statistical characteristics of air temperature variation within the shelterbelt network

In terms of shelterbelt grade (**Table 7**), the RII values of Grade I shelterbelts ranged from −0.066 to −0.061, those of Grade II shelterbelts ranged from −0.057 to −0.022, and those of Grade III shelterbelts were only −0.019 to 0.012. Among them, the average air temperature under Grade III shelterbelts reached 23.11 °C at the boll-opening stage, showing a warming phenomenon. In terms of spatial distance, air temperature generally showed a trend of first increasing and then stabilizing with increasing distance from the shelterbelt. Air temperature was lowest at 0.5H, gradually increased at 1H, 2H, and 3H, and decreased slightly at 5H. For example, under Grade I shelterbelts at the seedling stage, air temperature increased from 28.85 °C at 0.5H to 34.72 °C at 3H, and then decreased to 33.36 °C at 5H. In terms of growth stages, air temperature decreased as the growth stage progressed. Temperature was highest at the seedling stage, with average values of 31.97 °C, 32.54 °C, and 35.08 °C under Grade I, Grade II, and Grade III shelterbelts, respectively, and was lowest at the boll-opening stage, with values of 19.88 °C, 21.59 °C, and 23.11 °C, respectively. The cooling effect was most stable from the budding stage to the flowering stage.

**Table 7:** Average air temperature at different horizontal distances and relative interaction index within farmland shelterbelts of different grades.

Farmland shelterbelt grade	Measurement period	Air temperature (°C) at different horizontal distances (H)						CK	RII
		0.5H	1H	2H	3H	5H	Average		
I	Seedling Stage	28.85	31.06	31.84	34.72	33.36	31.97	36.46	-0.066
	Budding Stage	27.26	26.94	28.05	28.27	28.71	27.85	31.45	-0.061
	Flowering Stage	24.02	26.80	27.49	28.04	28.81	27.03	30.79	-0.065
	Boll Opening Stage	19.08	19.41	20.01	20.19	20.70	19.88	22.58	-0.064
II	Seedling Stage	30.55	31.67	33.03	33.31	34.14	32.54	36.46	-0.057
	Budding Stage	27.56	28.53	29.04	30.16	29.89	29.03	31.45	-0.040
	Flowering Stage	25.94	28.22	28.76	29.85	29.86	28.53	30.79	-0.038
	Boll Opening Stage	20.52	21.31	21.76	21.93	22.43	21.59	22.58	-0.022
III	Seedling Stage	32.85	34.37	35.43	36.02	36.71	35.08	36.46	-0.019
	Budding Stage	28.53	29.97	30.81	31.64	32.17	30.63	31.45	-0.013
	Flowering Stage	28.24	30.08	31.02	31.59	31.97	30.58	30.79	-0.003
	Boll Opening Stage	22.77	22.61	23.17	23.10	23.91	23.11	22.58	0.012

#### 3.5.2 Spatiotemporal variation of air temperature within the shelterbelt network

**Figure 7:**
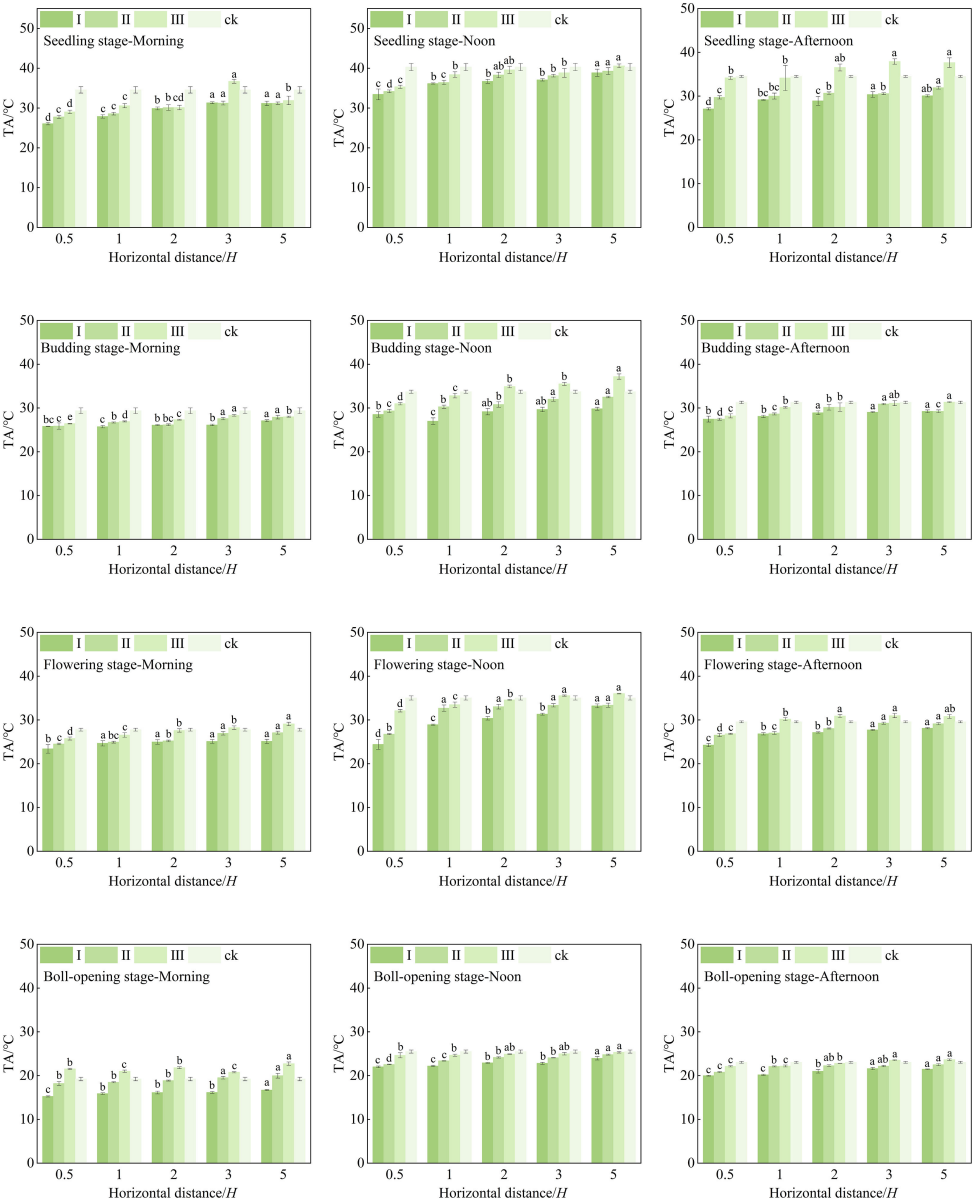
Variation in air temperature with horizontal distance and observation period within farmland shelterbelts of different grades.

In terms of shelterbelt grade (**Figure 7**), the overall air temperature followed the order Grade I < Grade II < Grade III < CK. In terms of spatial distance, air temperature was lowest at 0.5H under all shelterbelt types. As the distance extended to 1H, 2H, 3H, and 5H, air temperature gradually increased and approached the CK level at 5H (**Table 8**). Among them, Grade I shelterbelts showed the largest temperature difference between 0.5H and 5H, while Grade III shelterbelts showed the smallest difference. In terms of growth stages, air temperature showed a decreasing trend as the growth period progressed, and diurnal differences were obvious. The overall temperature was highest at the seedling stage, followed by the flowering stage, and lowest at the boll-opening stage. Within the same growth stage, the temperature at noon was higher than that in the morning and evening, and the cooling effect was most evident at noon. Grade I shelterbelts showed the greatest cooling amplitude at noon during the seedling stage, with a relatively large cooling amplitude compared with CK, whereas Grade III shelterbelts showed almost no cooling effect in the evening during the boll-opening stage, with air temperature basically consistent with CK.

**Table 8:** One-way ANOVA results for air temperature among horizontal distances within farmland shelterbelts of different grades.

Measurement period		Statistics	Morning			Noon			Afternoon		
			I	II	III	I	II	III	I	II	III
Seedling Stage		F_(df1, df2)_	F_(4,40)_=53.47	F_(4,40)_=8.27	F_(4,40)_=54.93	F_(4,40)_=9.55	F_(4,40)_=12.40	F_(4,40)_=6.40	F_(4,40)_=6.25	F_(4,40)_=4.65	F_(4,40)_=5.42
		p	<0.001	<0.001	<0.001	<0.001	<0.001	<0.001	<0.001	<0.004	<0.001
Budding Stage		F_(df1, df2)_	F_(4,40)_=3.40	F_(4,40)_=8.70	F_(4,40)_=15.10	F_(4,40)_=9.02	F_(4,40)_=16.51	F_(4,40)_=29.84	F_(4,40)_=4.12	F_(4,40)_=9.90	F_(4,40)_=7.77
		p	<0.017	<0.001	<0.001	<0.016	<0.002	<0.001	<0.007	<0.001	<0.001
Flowering Stage		F_(df1, df2)_	F_(4,40)_=2.92	F_(4,40)_=6.92	F_(4,40)_=11.26	F_(4,40)_=44.58	F_(4,40)_=10.55	F_(4,40)_=23.66	F_(4,40)_=35.44	F_(4,40)_=3.41	F_(4,40)_=17.84
		p	<0.033	<0.001	<0.001	<0.001	<0.001	<0.001	<0.001	<0.017	<0.001
Boll Opening Stage		F_(df1, df2)_	F_(4,40)_=5.80	F_(4,40)_=6.76	F_(4,40)_=15.96	F_(4,40)_=8.90	F_(4,40)_=38.68	F_(4,40)_=5.02	F_(4,40)_=9.63	F_(4,40)_=9.63	F_(4,40)_=22.22
		p	<0.001	<0.001	<0.001	<0.001	<0.001	<0.002	<0.001	<0.001	<0.001

The results indicate the significance of differences in air temperature among horizontal distances (0.5H, 1H, 2H, 3H, and 5H) within the same shelterbelt grade, growth stage, and observation period. F represents the one-way ANOVA statistic, df1 and df2 represent the between-group and within-group degrees of freedom, respectively, and p represents the significance level.

### 3.6 Principal component analysis of microclimate factors

**Figure 8:**
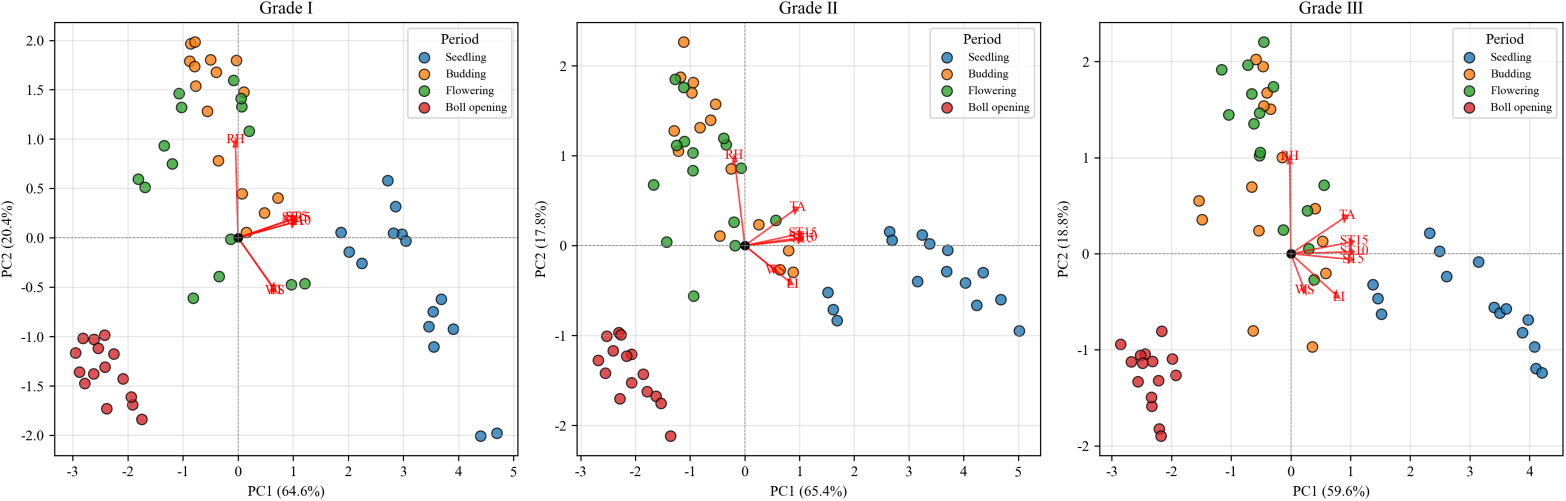
Principal component analysis of microclimate characteristics within farmland shelterbelts of different grades.

The PCA results showed that the microclimate characteristics of shelterbelts with different grades differed obviously across different growth stages (**Figure 8**). The cumulative explanatory rates of the first two principal components for Grade I, Grade II, and Grade III shelterbelts were 85.0%, 83.2%, and 78.4%, respectively, indicating that the first two principal components could effectively reflect the variation characteristics of microclimate. Among them, the explanatory rates of PC1 were 64.6%, 65.4%, and 59.6%, respectively, while those of PC2 were 20.4%, 17.8%, and 18.8%, respectively. Samples from different growth stages showed relatively obvious separation characteristics in the ordination space. Samples from the seedling stage were mainly distributed in the positive direction of PC1, samples from the boll-opening stage were mainly distributed in the negative direction of PC1, while samples from the budding and flowering stages were mostly distributed in the positive direction of PC2, indicating that the microclimate characteristics differed obviously among different growth stages. Grade I shelterbelt samples showed a higher degree of separation, whereas samples from some periods of Grade III shelterbelts showed a certain degree of overlap. The environmental factors showed certain correlations with each other. RH was mainly located in the positive direction of PC2, whereas WS and LI were mainly located in the negative direction of PC1 or PC2, indicating a negative correlation between air humidity and wind speed and light intensity. TA was close in direction to ST5, ST10, and ST15, indicating a strong synchronous variation between air temperature and soil temperature at different depths. The directions of soil temperature at different depths were generally consistent, indicating that their variation trends were relatively similar.

## 4 Discussion

From the overall results, shelterbelts with different structural configurations showed obvious differences in their regulation of farmland microclimate and exhibited clear spatiotemporal heterogeneity (Prakash et al., 2025). Among them, Grade I shelterbelts showed the best effects in wind-speed reduction, humidification, cooling, and light intensity attenuation due to their greater number of rows, complete belt continuity, moderate porosity, and greater tree height, while Grade II shelterbelts were at an intermediate level. Grade III shelterbelts had obviously weakened microclimate regulation capacity due to broken belts, single-row configuration, and excessive porosity. This indicates that shelterbelt structural configuration is an important factor affecting farmland microclimate (Baker et al., 2021), and further suggests that a reasonable belt structure can form a stable low-wind-speed airflow zone, thereby affecting light distribution, water vapor diffusion, and heat exchange processes. In contrast, high porosity and broken-belt structures can enhance airflow penetration and weaken the ability to form a stable microenvironment within the shelterbelt network (Kurose et al., 2002). 

In terms of wind-speed regulation, this study found that all three grades of shelterbelts formed an obvious low-wind-speed zone at 0.5H, and wind speed then gradually recovered with increasing horizontal distance, indicating that the near-shelterbelt area is the core zone of the windbreak effect. This is mainly because the shelterbelt blocks incoming airflow and reduces near-surface momentum exchange, thereby forming a low-speed buffer zone behind the shelterbelt (Bao et al., 2012). Meanwhile, the effective influence range of Grade I shelterbelts was obviously larger than that of Grade II and Grade III shelterbelts. Due to its greater number of rows, porosity of 30%–40%, and continuous canopy structure, Grade I shelterbelts had a more compact structural configuration. When airflow passed through the shelterbelt, part of its kinetic energy was weakened by the frictional resistance provided by branches, leaves, and trunks (Vigiak et al., 2003), which was conducive to maintaining a stable low-speed zone, consistent with the results of (Ma et al., 2010). The relative wind-speed reduction rate of Grade III shelterbelts clearly decreased at the flowering and boll-opening stages, especially with rapid airflow recovery in some areas, indicating that broken-belt structures allowed airflow to penetrate through gaps and made it difficult to form a continuous and stable protected zone. This is similar to the findings of (Wang et al., 2024) and (Cheng et al., 2020). In addition, as the cotton growth period progressed, cotton canopy height and leaf area index gradually increased, and the shelterbelt and crop canopy formed a synergistic effect (Gonçalves et al., 2021), enhancing near-surface airflow obstruction. Therefore, the overall wind-speed reduction effect was strongest at the budding and flowering stages.

Changes in LI indicated that shelterbelts mainly regulated the light environment within the shelterbelt network through shading effects. Grade I shelterbelts showed the strongest LI attenuation capacity across all growth stages. This was because the number of shelterbelt rows, tree species composition, and porosity of Grade I shelterbelts directly affected their shading area. In addition, the greater mean tree height of Grade I shelterbelts resulted in a longer shading distance, affecting light penetration and airflow movement within the shelterbelt (Decocq et al., 2004). In contrast, LI under Grade III shelterbelts was even close to or higher than CK in some periods, indicating that excessive porosity and discontinuous canopy structure allowed light to pass through gaps in the shelterbelt, clearly weakening the shading effect, which is consistent with the results of (Popic et al., 2025). LI was lowest within the 0.5H–1H range under all shelterbelt grades and gradually recovered after 2H, indicating that the direct shading effect of the tree canopy was mainly concentrated in the near-shelterbelt area, similar to the findings of (Strobl et al., 2011). Meanwhile, LI increased with height in the vertical direction, and the middle and lower layers at 30–60 cm were most strongly shaded, especially at the budding and flowering stages. This was mainly because the cotton canopy gradually closed after cotton entered the rapid growth stage, and crop leaves formed secondary shading of light, causing the shelterbelt and crop canopy to jointly affect the internal light environment of the shelterbelt network (de Castro et al., 2014). Therefore, the distribution of LI within the shelterbelt network was influenced not only by shelterbelt structure but also by crop canopy structure (Hong et al., 2022). In addition, shelterbelts may affect aerosol movement and light scattering processes by changing wind-speed conditions, resulting in differences in light intensity distribution (Jinyuan et al., 2003). Although moderate shading can reduce high-temperature stress, excessive shading may reduce crop photosynthetic efficiency. Therefore, shelterbelt structural configuration needs to maintain a balance between protective benefits and light use (Childs and Flint, 1987).

The soil temperature results showed that shelterbelts can jointly alter the soil thermal environment through wind protection, shading, and humidification (Zhe et al., 2024; Han et al., 2025). The soil cooling effect of shelterbelts was reflected in the fact that the soil temperature in the 10–15 cm layer was consistently lower than that in the shallow soil layer, indicating that deeper soil was less affected by external climatic fluctuations, which is similar to the results of (Han et al., 2025). Spatially, a relatively low-temperature center formed in the 2H–3H area. The cooling and humidifying effects of shelterbelts changed heat exchange at the soil surface and reduced the direct effect of solar radiation on the soil (Tianpeng et al., 2022), allowing soil temperature to remain at a more stable level. In local areas under Grade III shelterbelts, soil warming even occurred at the boll-opening stage, indicating that when broken belts affected their shading and humidification capacity, solar radiation could directly act on the ground surface and accelerate soil heat exchange (Xiufen et al., 2021). In addition, soil temperature continuously decreased with the progression of the cotton growth period, consistent with the trend of regional seasonal air temperature changes (Potashkina and Koshelev, 2022). Grade I shelterbelts could slow soil temperature fluctuations, indicating that a reasonable structural configuration can improve the stability of the farmland thermal environment (Gao et al., 2024).

The results of air humidity and air temperature further indicated that the microclimate regulation of shelterbelts had an obvious synergistic effect. Shelterbelts increase near-surface aerodynamic roughness and weaken near-surface momentum transport and turbulent exchange, thereby reducing airflow transport capacity (Wang and Takle, 1997). Grade I shelterbelts showed the highest humidity and lowest temperature across all growth stages. Shelterbelts with continuous structure and moderate porosity allowed airflow to gradually dissipate kinetic energy while passing through the canopy and form a stable low-wind-speed zone behind the shelterbelt. After wind speed decreased, the diffusion of water vapor generated by shelterbelt and crop transpiration weakened, which was conducive to the accumulation of air humidity (Xie and Li, 2001). Meanwhile, Canopy structure affects the surface energy balance by altering the process of radiative attenuation. The interception of incoming shortwave radiation by the tree canopy reduces net radiation input, thereby shifting a greater proportion of energy toward latent heat flux and indirectly enhancing evaporative cooling effects. Meanwhile, reduced wind speed further suppresses water vapor diffusion, causing water vapor released through crop transpiration and soil evaporation to be retained within the shelterbelt system, ultimately forming a relatively high-humidity microclimate. At the same time, air temperature was lowest at 0.5H and then gradually increased with distance. The synergistic effect of shelterbelt shading and humidification greatly reduced heat input from solar radiation, indicating that the near-shelterbelt area was most strongly affected by shading and evaporative cooling (Jacobs et al., 2022).

The PCA results showed that different grades of shelterbelts differed obviously in their regulation of farmland microclimate, and that there were strong coupling relationships among microclimate factors. The directional relationships of environmental factors in the ordination space indicated that microclimate factors did not vary independently. RH was generally opposite in direction to WS and LI, indicating that reduced wind speed and weakened light intensity were conducive to the accumulation of water vapor in the air. TA was close in direction to ST5, ST10, and ST15, indicating a strong synchronous variation between air temperature and soil temperature. Previous studies have pointed out that shelterbelts can form a low-wind-speed and high-humidity microclimate environment by weakening near-surface airflow exchange, reducing evapotranspiration consumption, and changing light distribution processes (Abdalazeem et al., 2024). Meanwhile, moderate porosity is more conducive to forming a stable protective effect. When shelterbelt porosity reaches 25%–40%, its comprehensive microclimate regulation capacity is usually stronger (Gao, 2010). In this study, Grade I samples showed a higher degree of separation, while samples from some periods of Grade III showed overlap, further indicating that high porosity and broken-belt structures weaken the integrated regulation of shelterbelts on wind speed, light intensity, and temperature–humidity conditions.

In addition, shelterbelt-induced microclimate regulation is not entirely positive. Strong wind protection, cooling, and humidification effects are beneficial for alleviating high-temperature and evapotranspiration stress in farmland in arid regions, but excessive shading may reduce the light available to crops. This is especially important during the cotton seedling stage and rapid growth stage, when an excessive decrease in LI may affect photosynthesis. In this study, Grade I shelterbelts showed better performance, indicating that under the conditions of arid cotton fields in Xinjiang, shelterbelt structures with good continuity and moderate porosity are more conducive to forming a stable microclimate. However, the results of this study were mainly based on the arid oasis cotton field system in Alar and should not be directly extended to other regions. Future studies should further verify the effects of shelterbelts on microclimate regulation by considering regional climatic conditions, crop types, and other factors.

## 5 Conclusion

This study showed that shelterbelts with different structural configurations had obviously different regulatory capacities for farmland microclimate. Overall, Grade I shelterbelts performed better than Grade II and Grade III shelterbelts in wind-speed reduction, humidification, cooling, and reducing light intensity (LI). Their microclimatic effects were mainly concentrated within the range of 0.5H–3H behind the shelterbelt. Among them, wind-speed reduction was most obvious near 0.5H, air humidity was higher near 1H, and LI and air temperature decreased more obviously in the near-shelterbelt area, indicating that shelterbelts with better continuity and moderate porosity were more likely to form a stable microclimate buffer zone. In contrast, due to broken belts and higher porosity, Grade III shelterbelts showed obviously weakened windbreak and hydrothermal regulation capacities, and the environmental conditions in some periods gradually approached the CK level.

The microclimatic effects of shelterbelts showed obvious dynamic changes across different growth stages. At the seedling stage, the cotton canopy was relatively small, and microclimate variation was mainly affected by shelterbelt structure. At the budding and flowering stages, with the rapid growth of cotton, the combined effects of shelterbelts and the crop canopy formed a relatively stable low-wind-speed and high-humidity environment within the shelterbelt network, and the protective effect was most obvious at this stage. At the boll-opening stage, due to plant senescence and canopy degradation, some microclimate factors gradually recovered to the state of open farmland. The PCA results further showed that the cumulative explanatory rates of the first two principal components for Grade I, Grade II, and Grade III shelterbelts were 85.0%, 83.2%, and 78.4%, respectively, which could effectively reflect the variation characteristics of farmland microclimate. Among them, RH was opposite in direction to WS and LI, while TA was close in direction to ST5, ST10, and ST15, indicating obvious coupling relationships among wind speed, light intensity, temperature, and air humidity. Shelterbelts further affected the hydrothermal environment within the shelterbelt network by changing the airflow transport process.

Overall, shelterbelts do not regulate the farmland environment through a single factor, but jointly form a microclimate system with coordinated changes in wind, light, heat, and moisture by affecting the wind-speed field, light transmission, and heat exchange. Under the conditions of arid oasis cotton fields in Xinjiang, shelterbelts with better continuity and moderate porosity are more conducive to alleviating climatic stresses such as high temperature, drought, and strong wind, and are of great significance for maintaining the stability of the farmland ecological environment. Therefore, in the construction of farmland shelterbelts in arid regions, attention should be paid to the integrity of shelterbelt structure and reasonable porosity configuration, so as to improve the ability of agroforestry systems to cope with high-temperature, drought, and wind-sand stresses.

## Data Availability

The data analyzed in this study is subject to the following licenses/restrictions: This dataset was derived from field observations in the Alar Reclamation Area, Xinjiang, China, under a typical cotton agroforestry system. The microclimate data are limited to four key cotton growth stages and specific weather conditions without rainfall or irrigation within 10 days before sampling. The results apply only to farmland shelterbelts in arid desert–oasis ecotones with similar stand structure, tree species, and climatic background. They cannot be directly extrapolated to other climate zones, crop types, shelterbelt species compositions, or soil–hydrological conditions without further validation. Requests to access these datasets should be directed to lixiaoqian1@xjshzu.com.
